# Creatine Supplementation and Brain Health

**DOI:** 10.3390/nu13020586

**Published:** 2021-02-10

**Authors:** Hamilton Roschel, Bruno Gualano, Sergej M. Ostojic, Eric S. Rawson

**Affiliations:** 1 Applied Physiology & Nutrition Research Group, Rheumatology Division, School of Physical Education and Sport, Faculdade de Medicina FMUSP, Universidade de Sao Paulo, Sao Paulo 01246-903, Brazil; gualano@usp.br; 2 Food Research Center, University of São Paulo, Sao Paulo 05508-080, Brazil; 3 FSPE Applied Bioenergetics Lab, University of Novi Sad, 21000 Novi Sad, Serbia; sergej.ostojic@chess.edu.rs; 4 Department of Health, Nutrition, and Exercise Science, Messiah University, Mechanicsburg, PA 17055, USA; erawson@messiah.edu

**Keywords:** phosphorylcreatine, dietary supplement, cognition, brain injury, concussion

## Abstract

There is a robust and compelling body of evidence supporting the ergogenic and therapeutic role of creatine supplementation in muscle. Beyond these well-described effects and mechanisms, there is literature to suggest that creatine may also be beneficial to brain health (e.g., cognitive processing, brain function, and recovery from trauma). This is a growing field of research, and the purpose of this short review is to provide an update on the effects of creatine supplementation on brain health in humans. There is a potential for creatine supplementation to improve cognitive processing, especially in conditions characterized by brain creatine deficits, which could be induced by acute stressors (e.g., exercise, sleep deprivation) or chronic, pathologic conditions (e.g., creatine synthesis enzyme deficiencies, mild traumatic brain injury, aging, Alzheimer’s disease, depression). Despite this, the optimal creatine protocol able to increase brain creatine levels is still to be determined. Similarly, supplementation studies concomitantly assessing brain creatine and cognitive function are needed. Collectively, data available are promising and future research in the area is warranted.

## 1. Introduction

The ergogenic effects of creatine supplementation are well documented, with evidence supporting its efficacy in increasing muscle strength, lean mass, and exercise performance/muscle function, particularly when combined with exercise in different populations, from athletes to a wide spectrum of patient populations [1,2,3].

Creatine mechanisms of action involve rapid energy provision by transferring the N-phosphoryl group from phosphorylcreatine (PCr) to adenosine diphosphate (ADP), thus resynthesizing adenosine triphosphate (ATP) and spatial energy buffering, transferring energy from the mitochondria to the cytosol. These mechanisms are responsible for facilitating ATP homeostasis during high energy turnover, maintaining a low ADP concentration and reducing Ca^2+^ leakage from the sarcoplasmic reticulum and impairment of force output of the muscle [4,5,6]. Additionally, creatine could also attenuate the formation of reactive oxygen species by its coupling with ATP into the mitochondria or by scavenging radical species in an acellular setting [7]. Its direct and indirect antioxidant effects have been suggested to have therapeutic effects in neurodegenerative diseases [8].

Although most of the total body’s creatine is found in skeletal muscle, the brain is also a very metabolically active tissue, accounting for up to 20% of the body’s energy consumption [9,10]. Creatine kinase (CK), a main enzyme involved in the ATP/CK/PCr system, is also expressed in a brain-specific isoform (BB-CK) [4,5,6], suggesting that creatine may also be relevant for energy provision to the central nervous system (CNS). In fact, creatine-deficient syndromes involving brain creatine depletion are characterized by major mental and developmental disorders (e.g., mental retardation, learning delays, autism, and seizures), which may be partially reversed by creatine supplementation [11,12,13,14]. Cognitive processing may also be affected by creatine metabolism, as it may facilitate ATP homeostasis during periods of rapid or altered brain ATP turnover, such as during complex cognitive tasks, hypoxia, sleep deprivation, and some neurological conditions [3,15,16]. Additionally, creatine supplementation might be beneficial for mild traumatic brain injury (mTBI), which is also associated with changes in brain energy needs. The effects of creatine supplementation on brain creatine levels, cognitive processing, and mTBI have been previously reviewed [3,17,18]. As this is a growing field, the purpose of this short review is to provide an update regarding the effects of creatine supplementation on brain health in humans beyond what is discussed in Dolan et al. [3].

## 2. The Effects of Creatine Supplementation on Brain Creatine Levels

**Figure 1:**
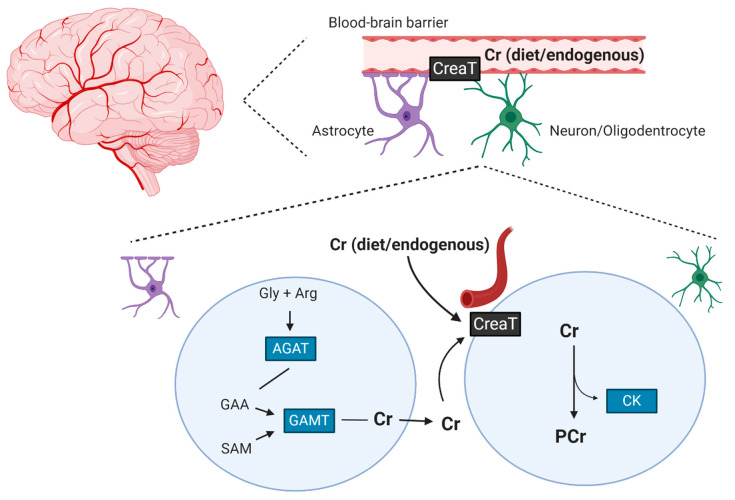
Dietary creatine is transported through the blood–brain barrier via a creatine transporter. Astrocytes cells can also endogenously produce creatine, which is taken up by the neurons expressing the creatine transporter. Cr: creatine; PCr: phosphocreatine; Gly: glycine; Arg: arginine; AGAT: L-ariginine: glycine amidinotransferase; GAA: guanidinoacetate; GAMT: guanidinoacetate methyltransferase, SAM: S-adenosylmethionine; CreaT: Cr transporter. Created with BioRender.com.

While muscle exclusively relies on dietary ingestion and endogenous synthesis from the liver, kidneys, and pancreas [19], the brain can synthesize creatine. The enzymatic apparatus necessary for endogenous creatine synthesis is found in the nervous system and creatine transporters are found at the blood–brain barrier, neurons and oligodendrocytes cells, indicating that brain creatine may not solely dependent on endogenous production from other organs or dietary sources [20]. Furthermore, brain creatine seems not to be influenced by habitual dietary intake from food, as similar brain PCr is found between vegetarians and omnivores [21]. Still, if the intracerebral synthesis is limited due to inherited disorders of creatine-catalyzing enzyme(s) machinery, dietary provision of the compound can positively affect brain creatine concentrations [22]. Figure 1 illustrates endogenous creatine synthesis in the brain and its transport across the blood–brain barrier.

Brain creatine content has been suggested to be affected by other factors, such as aging [23]; however, comparable levels of brain PCr have also been found between apparently healthy elderly and young individuals [24]. Other factors related to aging that may influence brain creatine concentrations include reduced brain and/or physical activity, depression, schizophrenia, and panic disorder. The overlap between these factors may be misleading as to what might be identified as an age-related decline (reviewed in Rawson and Venezia [25]).

While consistent information is available on supplementation protocols aimed at increasing muscle creatine content [26], much less is known regarding the optimal supplementation strategy to increase brain creatine levels. A large heterogeneity in respect to brain creatine assessment technique (i.e., total brain creatine as assessed by H^1^-NMR vs. brain PCr as assessed by P^31^-NMR), supplement dose and duration (range 2 to 20 g/d for 1 to 8 weeks), and population characteristics (including habitual dietary creatine intake, health status, etc.) hampers direct comparison between the few studies on the topic. Further confusion is introduced by the fact that creatine content may differ regionally within the brain [25,27]. Nevertheless, collectively, the available literature suggests possible increases in both creatine and PCr in the brain following supplementation, though smaller than that seen in muscle (~half the increase) [3]. As reviewed in detail by Dolan et al. [3], there are currently 12 studies of the effects of creatine supplementation on brain creatine or PCr concentrations. Nine of these studies showed a significant increase in brain creatine, averaging about 5 to 10%, which is less than the increase in muscle creatine or PCr resulting from similar supplementation protocols. Some of these studies focused on patient populations who have altered brain energetics, including females with major depressive disorder, depression and amphetamine use, and selective serotonin uptake inhibitor resistant depression. Other groups investigated the effects of creatine ingestion on brain creatine levels in apparently healthy individuals. There is no clear indication why a small number of studies were ineffective at increasing brain creatine despite using similar supplementation protocols, but differences in baseline brain creatine levels, brain creatine assessment, population characteristics, and dosing strategies likely play a role.

The explanation for these differences in creatine uptake between muscle and brain remains speculative. As discussed, brain creatine content may rely less on exogenous creatine than muscle [20,21,24,28], which could theoretically involve a down-regulated response in brain creatine synthesis upon supplementation. Alternative to this hypothesis is the demonstration that the brain lacks the expression of creatine transporter in the astrocytes involved in the blood–brain barrier, thus implying a limited permeability of the brain to the circulating creatine [29], which is in line with the lack of increase in brain creatine following supplementation reported by some studies [24,28,30]. It is also plausible to speculate that if the brain is, in fact, resistant to exogenous creatine, a high-dose, long duration protocol would be needed, such as those used in the study by Dechent et al. [27] (i.e., 20 g/day for 4 weeks). The need for a higher supplementation dose in order to increase brain creatine level, as compared to the supplementation dose required for muscle, is further corroborated by data available from the only study assessing both muscle and brain creatine levels in response to supplementation, with increases found in the former, but not the latter [24]. Of interest, supplementing guanidinoacetic acid (GAA), a creatine precursor, was found superior to an equimolar dose of creatine in increasing brain creatine content [31]. While creatine is mainly transported via a specific transporter (SLC6A8 or CT1; also used for GAA transport), dietary GAA could be imported to the brain through additional delivery transporters and routes (including SLC6A6, GAT2, and passive diffusion) [32] and become readily available for methylation to creatine. Although preliminary, these data are of relevance considering the inherent capacity of the brain to synthesize creatine and its theoretical impaired ability to transport creatine through the blood–brain barrier, thus warranting further research on alternative strategies to increase brain creatine.

## 3. Creatine Supplementation and Cognition

The interest in the effects of creatine supplementation on cognition is not new. Despite the number of positive studies available on the subject (Summarized in Table 1), differences between investigations including study populations, cognitive function testing, and supplementation dosing and duration precludes direct comparison; however, some conclusions can be made. Although controversial [28,33,34], creatine supplementation may positively influence some aspects of cognition in different experimental paradigms [10,35,36,37,38,39,40]. Importantly, its effects seem more pronounced in stressful conditions such as hypoxia [8] and sleep deprivation combined with exercise [10,37,38]. Despite the suggestion that more complex or demanding cognitive processes are more prone to respond to supplementation (as they are more energy demanding), inconsistencies regarding cognitive test response to supplementation hampers further conclusions [37,38]), which may be attributed to differences in experimental design such as the sleep deprivation period and exercise intensity employed between studies.

In elderly individuals, specifically, literature is conflicting on the effects of creatine supplementation on cognitive performance. While McMorris et al. [16] showed improved cognitive performance, Alves et al. [33] found creatine (alone or associated with exercise training) ineffective. Both studies are limited by the lack of brain creatine concentration assessments, casting doubt on whether aging-related reduction in cognitive processing may arise from the presence of, for instance, neurodegenerative diseases or whether the supplementation protocol employed (designed for increasing muscle creatine content) may have been insufficient to significantly increase brain PCr. Recently, Smolarek et al. [43] found increased cognitive performance (and handgrip strength) after a 16 week intervention combining resistance training and creatine supplementation (5 g/day) in a pilot study including older adults. The results are, however, limited by the absence of an exercising control group and inconsistent cognitive performance in the control group across time, thus hampering further conclusion on the effects of supplementation alone.

It has been contended that vegetarians may differentially respond to creatine supplementation when compared to meat-eaters. In this respect, cognitive function has been shown to be improved in vegetarians after creatine supplementation [39]. Another study found greater effects on memory in vegetarians as compared to omnivores following creatine supplementation [15]. Importantly, the lack of a control group (meat-eaters) and the fact that between-group differences were due to decreased performance in the omnivores, rather than an improvement in the vegetarians, limits the conclusions of this study. Additionally, comparable brain creatine concentrations have been shown between meat-eaters and vegetarians [21], which undermines the theory that vegetarians should respond better than meat-eaters due to lower pre-supplementation brain creatine. More research should be conducted on the differential responses to creatine supplementation between vegetarians and omnivores.

Improvements in cognitive processing capability is also of interest to athletes. Several sports include motor control, decision making, coordination, reaction time, and other cognitive tasks as key aspects of performance, which may be affected by mental fatigue [45]. In this respect, creatine may play an ergogenic role, as, theoretically, it may mitigate mental fatigue, thus favouring performance. Indeed, creatine has been shown effective in attenuating the effects of sleep deprivation on throwing accuracy in rugby players [42], while no effect was observed on passing accuracy in non-stressed soccer players [46,47]. Brain creatine content was not assessed in these studies, raising uncertainty as to whether the results observed result from changes in brain creatine. Nonetheless, the discrepancy in the results may, at least partially, relate to the suggestion that creatine supplementation is most effective under stressed cognitive processes conditions such as sleep deprivation.

More recently, two studies revisited the subject, with interesting results. Borchio et al. [41] found improved performance in selected indexes of cognitive function after a time-trial track test in semi-professional mountain bikers supplemented with creatine. Interestingly, no prior cognitive deficit-inducing condition, such as sleep deprivation, was imposed, suggesting that creatine could potentially attenuate mental fatigue even in non-stressed situations. Van Cutsem et al. [44] studied the effects of creatine supplementation on mental fatigue and its negative effects on psychomotor skills in a non-athlete population and found that creatine was able to improve Stroop accuracy during a 90 min Stroop task and to increase strength endurance (assessed by a handgrip strength test) pre-to-post Stroop task. Importantly, no effects of supplementation were observed on the mental-fatigue-induced impairments in psychomotor and cognitive performance. Collectively, although these results suggest a potential role of creatine on mental fatigue, whether and to what extent this could affect specific sports performance remains to be elucidated.

## 4. Creatine Supplementation and Brain Injury, Concussion, and Hypoxia

One of the characteristics of traumatic brain injury is the alteration of ATP demand due to reduced blood flow and hypoxia [48]. Importantly, brain creatine is reduced following a mild traumatic brain injury (mTBI) [49], making creatine supplementation, and subsequent increase in brain creatine, a potentially valuable strategy to reduce severity of, or enhance recovery from, mTBI or concussion by offsetting negative changes in energy status. The duration of the dysregulation in brain energy metabolism is not clearly defined, but could remain for weeks if not years. Alosco et al. [50] reported on retired players from the National Football League (aged 40 to 69) who had experienced repetitive head impacts during their career and many years later had complaints of cognitive and/or behavioral/mood symptoms. In this cohort, there was a relationship between greater exposure to repetitive head impacts and decreased brain creatine in the parietal white matter. This indicates that there could be later-life derangements in brain energy metabolism subsequent to mTBI, and lends support to the concept that creatine supplementation could be valuable in enhancing recovery from mTBI, even years after the injury. In addition to its potential role in aiding the cellular energy crisis induced by injury, creatine may lessen other features of mTBI, such as membrane disruption leading to calcium influx, nerve damage, mitochondrial dysfunction, oxidative stress, and inflammation (reviewed in [48,51]).

In an experimental model mimicking the effects of mTBI, Turner et al. [10] found that supplementation was able to increase brain creatine and cognitive processing during oxygen deprivation. Animal models have also been employed to investigate the effects of creatine supplementation on traumatic brain injury. Sullivan et al. [52] found significant reduction in brain damage following traumatic brain injury in both mice (36%) and rats (50%). These large effects are compelling, but as humans only increase brain creatine about 10% in response to supplementation and some animals increase brain creatine 30 to 50%, it is difficult to generalize these data to the general population or athletes [53]. Despite its potential, experimental data in humans are still scarce; however, results from the few studies available are promising. Creatine supplementation has been shown able to improve cognition, communication, self-care, personality, and behavior, and reductions in headaches, dizziness, and fatigue in children with mTBI [54,55].

Collectively, despite limited data, creatine supplementation seems potentially beneficial in reducing severity of or enhancing recovery from mTBI, warranting further studies on its role not only as a post-injury therapy but also as a neuroprotective agent in populations at high risk of mTBI. As has been described elsewhere, creatine supplements have documented muscular performance benefits, are inexpensive, widely available, and have a strong safety profile [26,56,57,58,59,60,61,62,63]. Encouraging supplementation to reduce damage from or enhance recovery from mTBI based primarily on animal and theoretical data in lieu of clinical trials would ordinarily be considered premature. However, in this instance, given the devastating effects of mTBI, combined with the large body of safety and efficacy creatine supplementation data, encouraging supplementation for populations who are at high risk for mTBI might be considered more prudent.

## 5. Conclusions and Future Directions

There is a potential for creatine supplementation to improve cognitive processing, especially in conditions characterized by brain creatine deficits, which could be induced by acute stressors (e.g., exercise, sleep deprivation) or chronic, pathologic conditions (e.g., creatine synthesis enzyme deficiencies, mTBI, aging, Alzheimer’s disease, depression).

However, at least three main gaps remain. First, it is important to determine the optimal creatine protocol able to increase brain creatine levels. So far, dose-response studies are lacking and protocols are heterogenous. Second, supplementation studies concomitantly assessing brain creatine levels and cognitive function are needed, as it may help establish causation for the effect of creatine supplementation on cognition. Third, the identification of novel conditions in which creatine supplementation may be more effective in improving cognitive function is warranted as creatine in a rested healthy brain has been shown to have a lessened effect.

## Figures and Tables

**Table 1:** Effects of creatine supplementation on cognitive performance.

Population	Creatine Supplementation Protocol	Cognitive Tests (CT) Outcomes (O)	Reference
Healthy older women	20 g/day + 5 g/day for 24 weeks	CT: Mini-mental state examination, stroop, trail making, digit span, delay recall test and the short version of the geriatric depression scale O: No change	Alves et al. (2013) [33]
Semiprofessional, non-vegetarian, male mountain bikers	20 g/day for 7 days	CT: Simple and choice reaction time, differentiation task test, Eiksen flanker test and Corsi block test O: Creatine increased performance in choice reaction time, Eiksen flanker test and Corsi block test.	Borchio et al. (2020) [41]
Healthy young women (vegetarian and meat-eaters)	20 g/day for 5 days	CT: Word recall, simple and choice reaction time, rapid visual information processing and controlled oral word association test O: Word recall test performance was reduced in meat-eater after creatine supplementation (within-group comparison). Post supplementation performance was higher in vegetarians than in meat-eaters.	Benton and Donohoe (2011) [15]
Professional male rugby players who were sleep-deprived (3–5 h)	0.05 or 0.1 g/kg/bw for 1 day	CT: Rugby passing skill test O: Sleep deprivation reduced passing accuracy and creatine reversed this effect (trend for greater effect with larger dose).	Cook et al. (2011) [42]
Healthy young adults	20 g/day for 5 days + 5 g/day for 2 days	CT: Backward digit span test and ravens advanced progressive matrices. O: Backward digit span performance was increased after creatine.	Hammett et al. (2010) [35]
Healthy young men and women	5 g/day for 15 days	CT: Memory scanning, number-pair matching, sustained attention, arrow flankers and IQ test O: Aspect of improvement was reported in all the cognitive tests performed in the creatine group.	Ling et al. (2009) [36]
Healthy young men and women who were sleep-deprived (24 h)	20 g/day for 7 days	CT: Random number generation, forward and backward recall, visual reaction time, static balance and mood state O: Performance reduction was attenuated in the creatine group for random movement generation, choice reaction time, balance and mood.	McMorris et al. (2006) [38]
Healthy elderly men and women	20 g/day for 7 days	CT: Random number generation, forward and backward recall and long-term memory tests O: Forward number recall, forward and backward spatial recall and long-term memory performance were enhanced after creatine supplementation.	McMorris et al. (2007a) [16]
Healthy young men who were sleep-deprived (36 h)	20 g/day for 7 days	CT: Random number generation, short-term number recall, visual reaction time, cognitive effort, dynamic balance test and mood state O: Performance on the random number generation test was improved following creatine.	McMorris et al. (2007b) [37]
Healthy male and female children	0.3 g/kg/day for 7 days	CT: Stroop, Rey auditory verbal learning test, Raven progressive matrices and trail making test O: No change	Merege-Filho et al. (2017) [28]
Vegan and vegetarian healthy male and female young adults	5 g/day for 6 weeks	CT: Ravens advanced progressive matrices and Wechsler auditory backward digit span task O: Creatine improved performance on the Raven’s test and the backward digit span task.	Rae and Broer (2015) [17]
Healthy male and female young adults	0.03 g/kg/day for 6 weeks	CT: Automated neuropsychological assessment metrics O: No change	Rawson et al. (2008) [34]
Male and female institutionalized older adults (with full physical and mental capacities preserved)	5 g/day for 16 weeks	CT: Montreal Cognitive Assessment (MoCA) questionnaire O: Creatine (plus resistance training) improved MoCA scores.	Smolarek et al. (2020) [43]
Healthy male and female young adults exposed to experimental hypoxia	20 g/day for 7 days	CT: Neuropsychological test comprising verbal and visual memory, finger tapping, symbol digit coding stroop test, test of shifting attention, continuous performance test, alertness and peripheral and corticomotor excitability O: Creatine supplementation offset hypoxia-induced decrements in a number of cognitive tests.	Turner et al. (2015) [10]
Healthy male and female young adults exposed to mental fatigue (90 min Stoop task)	20 g/day for 7 days	CT: Psychomotor performance (visuomotor task with Fitlight-hardware and software), strength endurance task, Flanker test, heart rate, blood glucose, success motivation and intrinsic motivation, mood, session ratings of perceived exertion and mental fatigue O: Accuracy throughout the 90 min Stroop task and strength endurance (in the non-dominant hand) were improved with creatine. No other effects of creatine supplementation were observed.	Van Cutsem et al. (2020) [44]
Healthy male and female young adults	8 g/day for 5 days	CT: Serial calculation task (Uchida-Kraeplin) O: Both groups increased mean performance. Mental fatigue, assessed during the second half of the test, was increased in the creatine group only.	Watanabe et al. (2002) [40]
